# Graded inhibition of oncogenic Ras-signaling by multivalent Ras-binding domains

**DOI:** 10.1186/1478-811X-12-1

**Published:** 2014-01-02

**Authors:** Martin Augsten, Anika Böttcher, Rainer Spanbroek, Ignacio Rubio, Karlheinz Friedrich

**Affiliations:** 1 Department of Oncology-Pathology, Karolinska Institutet, 171 76, Stockholm, Sweden; 2 Institute of Vascular Medicine, University Hospital Jena, Jena, Germany; 3 Center for Sepsis Control and Care, University Hospital Jena, Jena, Germany; 4 Institute of Molecular Cell Biology, University Hospital Jena, Jena, Germany; 5 Institute of Biochemistry II, University Hospital Jena, Jena, Germany; 6 Present address: German Research Center for Environmental Health, Neuherberg, Germany

**Keywords:** Cellular transformation, Ras, RBD, Tet-off system, MSOR: *m*ultivalent *s*cavengers of *o*ncogenic *R*as

## Abstract

**Background:**

Ras is a membrane-associated small G-protein that funnels growth and differentiation signals into downstream signal transduction pathways by cycling between an inactive, GDP-bound and an active, GTP-bound state. Aberrant Ras activity as a result of oncogenic mutations causes *de novo* cell transformation and promotes tumor growth and progression.

**Results:**

Here, we describe a novel strategy to block deregulated Ras activity by means of oligomerized cognate protein modules derived from the Ras-binding domain of c-Raf (RBD), which we named MSOR for *m*ultivalent *s*cavengers of *o*ncogenic *R*as. The introduction of well-characterized mutations into RBD was used to adjust the affinity and hence the blocking potency of MSOR towards activated Ras. MSOR inhibited several oncogenic Ras-stimulated processes including downstream activation of Erk1/2, induction of matrix-degrading enzymes, cell motility and invasiveness in a graded fashion depending on the oligomerization grade and the nature of the individual RBD-modules. The amenability to accurate experimental regulation was further improved by engineering an inducible MSOR-expression system to render the reversal of oncogenic Ras effects controllable.

**Conclusion:**

MSOR represent a new tool for the experimental and possibly therapeutic selective blockade of oncogenic Ras signals.

## Background

The prototypical Ras isoforms H-Ras, K-Ras and N-Ras (collectively Ras) are membrane-associated small G-proteins that cycle between an active, GTP-bound and an inactive, GDP-bound state. Ras becomes activated, that is GTP-loaded, by guanine nucleotide exchange factors (GEFs) such as Sos or RasGRP, which are themselves engaged and activated downstream of various cell surface receptors via adapter proteins, like Shc and Grb-2 and/or via second messenger lipids like phosphatidic acid or diacylglycerol [1,2]. Inactivation of GTP-loaded Ras occurs through a GTP-hydrolase (GTPase) activity intrinsic to Ras and enhancement of this reaction by GTPase activating proteins (GAPs) [1,3]. Ras function is also controlled by a series of obligatory post-translational modifications which include an initial farnesylation step and the reversible attachment of palmitate groups to N-Ras and H-Ras [4]. Although many details of this complex processing remain unknown, it is well established that the correct posttranslational processing is required to direct Ras to cellular membranes and specific microdomains within the plasma membrane (PM) [5].

Ras proteins play important roles in receptor-mediated signal transduction pathways that control cell proliferation and differentiation and are moreover critically involved in the regulation of cell motility and invasiveness [3,6,7]. Ras regulates these processes by feeding signals into various major signaling pathways, prominently the Erk kinase pathway, a cascade of protein kinases which ultimately drives the transcription of key target genes for cell cycle progression and other processes [8]. Ras-dependent activation of the Erk kinase pathway relies on the productive contact of Ras-GTP with members of the Raf family of serine/threonine kinases (collectively Raf), which together with other coincident inputs result in Raf activation [9,10]. Raf binds Ras-GTP via a N-terminally located Ras-binding domain (RBD), roughly 80 amino acid residues in size, that features several orders of magnitude higher affinity for Ras-GTP than Ras-GDP [11,12]. Several amino acid residues in the RBD are critical for the interaction with Ras-GTP and mutation of these sites impairs the high affinity binding of RBD to Ras-GTP [13,14].

Tight regulation of the Ras activation status is critical for cell physiology. Mutations that convert Ras into an oncoprotein are found in up to 25% of human tumors [15] (http://www.sanger.ac.uk). Oncogenic mutations, including substitutions of glycine 12 and glutamine 61, compromise the intrinsic and GAP-promoted GTPase activity of Ras. In agreement with a critical role of continuous aberrant Ras-GTP elicited signaling in oncogenesis, defects in GAP function or gain-of-function mutations in GEFs do also result in cell transformation and other pathological conditions [1,16-18]. Aberrant activation of the Ras/Raf-pathway contributes to essential aspects of tumor development and progression such as cell cycle deregulation, avoidance of apoptosis, cell motility and drug resistance and are moreover known to be important for tumor maintenance and cancer cell viability at late stages of tumorogenesis [19,20]. Due to its nodal role in cell transformation, Ras was early on identified as an attractive target for pharmaceutical intervention. Soon after the identification and characterization of farnesyl transferase (FTase) as the enzyme responsible for the first in the series of Ras-modifications, FTase inhibitors which efficiently blocked Ras mediated cell transformation in cell culture and animal models were developed [21-23]. However, the results of clinical trials with a large panel of FTase inhibitors were disappointing and discouraged many from pursuing further efforts to target oncogenic Ras. Later, Ras neutralizing antibodies were employed as oncogenic Ras blockers in cell culture experimentation [24-26] and mutant Ras epitopes were exploited for their suitability as antigens in the development of cancer vaccines [27]. Further approaches to target oncogenic Ras rested on antisense oligonucleotides directed to the Ras mRNA [28], and more recently on exploiting structural information and improved *in silico* approaches to identify and target druggable pockets or moieties that affect Ras nucleotide exchange [29,30], Ras activation [31,32], effector interaction [33,34] or binding to escort proteins critical for subcellular trafficking [35]. Moreover, numerous studies have targeted Ras downstream effector pathways such as Raf kinases, MEK or PI3Ks [36,37]. However, to date, Raf, MEK and PI3K inhibitors have shown little efficacy in the treatment of oncogenic Ras driven tumours, essentially evidencing that we still do not understand all intricacies of Ras signaling in the context of oncogenesis. In sum, in the light of the high prevalence of Ras mutations in human tumors it is sobering that 30 years after its discovery as the first human oncogene no strategy for the direct blockade of oncogenic Ras has reached clinical use.

In the present study we have developed and characterized a novel approach for the blockade of Ras-GTP dependent signaling. We demonstrate that oligovalent, Ras-GTP scavenging probes composed of up to 3 wild-type or mutant RBD modules, behave as “*m*ultivalent *s*cavengers of *o*ncogenic *r*as” (MSOR) that can be applied to inhibit various parameters of Ras-dependent oncogenic cell transformation in an adjustable fashion.

## Results

### Oligovalent Ras-binding domains block oncogenic Ras-induced signaling

**Figure 1:**
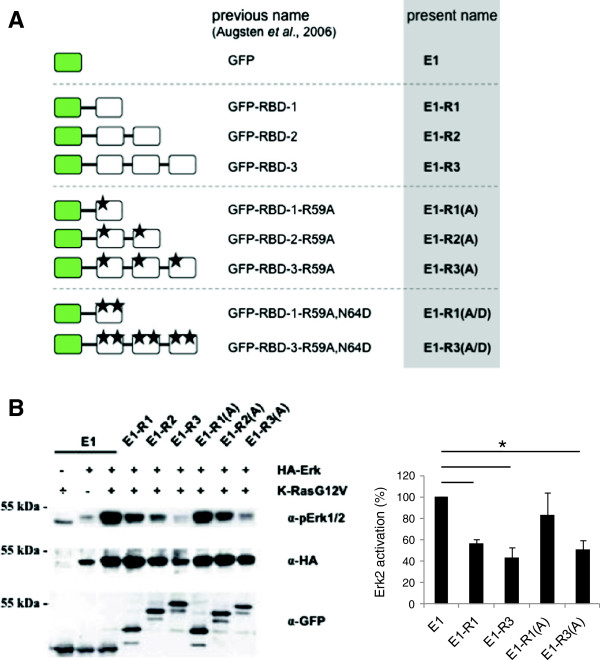
**MSOR inhibit oncogenic Ras-induced signaling. (A)** Schematic presentation of the EGFP-fused RBD mono- and oligomers explored in this study. The different mono- di and trivalent probes (R1, R2, R3) are composed of either wild-type or mutant c-Raf-derived RBDs. The RBD-mutations R59A (*) and R59A/N64D (**) are abbreviated by (A) and (A/D), respectively. Oligovalent probes consisting of two or three RBDs are collectively described as MSOR for *m*ultivalent *s*cavengers of *o*ncogenic *R*as. **(B)** The influence of RBD monomers and MSOR on Ras-induced signaling was studied in NIH3T3 cells transiently expressing K-RasG12V, HA-tagged Erk2 and mono-, di- or trivalent EGFP-RBDs (wild type or R59A-mutant). Cell lysates were subjected to western blot analysis detecting phosphorylated and total Erk2 and expression of EGFP-RBD-constructs. Signals from four independent experiments were quantified and expressed as ratio of phosphorylated and total Erk2.

We have previously employed modular probes consisting of oligomerized Ras-binding domain (RBD) units as novel Ras-GTP-specific probes. Fused to EGFP, these oligomers are instrumental for the visualization of growth factor-stimulated activation of endogenous Ras in live cells [38-41]. In the course of those studies we noticed that oligomeric RBD-variants sequestered Ras-GTP *in vitro* in an oligomerization grade-dependent fashion and interfered with Ras-dependent signaling in COS-7 cells [38]. This prompted us to test whether or not RBD-oligomers can be used to block the action of oncogenic Ras. In the present study we use the MSOR nomenclature introduced in ref. [39] which is recapitulated in Figure 1A.

In order to confirm the previously observed inhibitory effect of MSOR on oncogenic Ras-signaling we compared the impact of mono-, di-and trimeric wildtype RBDs (E1-R1, E1-R2, E1-R3, respectively) on oncogenic K-RasG12V induced Erk kinase activation in mouse fibroblasts. NIH3T3 cells were transfected with various combinations of constitutively active, oncogenic K-RasG12V, HA-tagged Erk2 and different RBD-expressing plasmids. As expected, K-RasG12V enhanced activation of the co-transfected Erk2 kinase (as assessed by Erk2 phosphorylation) and this activation was diminished in the presence of mono- and oligovalent wild-type RBD constructs (Figure 1B). Importantly, the blocking efficiency of RBDs increased as the degree of oligomerization rose from single (E1-R1) to triple (E1-R3) with the latter abolishing RasG12V-dependent signaling.

To substantiate this observation and to ascertain the specificity of the blocking effect, we tested RBD-variants containing the R59A mutation which lowers the affinity of RBD for Ras-GTP by about 30fold [14,42]. This type of mutations is commonly used in the context of full-length Raf to disrupt Ras-to-Raf signal propagation in cell biological studies [11]. In line with its inability to interact with Ras-GTP *in vitro*[38] the RBD-R59A-monomer E1-R1(A) did not significantly block Ras-K-RasG12V-induced phosphorylation of Erk2 (Figure 1B). However, expression of the same RBD-R59A module as a dimer (E1-R2(A)) or trimer (E1-R3(A)) inhibited RasG12V-induced signaling with gradually increasing strength, albeit always with lower potency than the wild-type MSOR counterparts. Noteworthy, E1-R3(A) expression was lower than that of its monomeric counterpart E1-R1(A), arguing that the gradual increase in blocking strength did not reflect the mere increase in numbers of RBD modules but rather was contingent on the presence of concatenated RBD units. These data recapitulated previous findings from COS-7 cells [38], and illustrated the validity of the oligomerization principle as a means to raise and tune the avidity and affinity of oligovalent binding domains for Ras-GTP.

### RBD-oligomers inhibit different parameters of Ras-mediated cellular transformation

**Figure 2:**
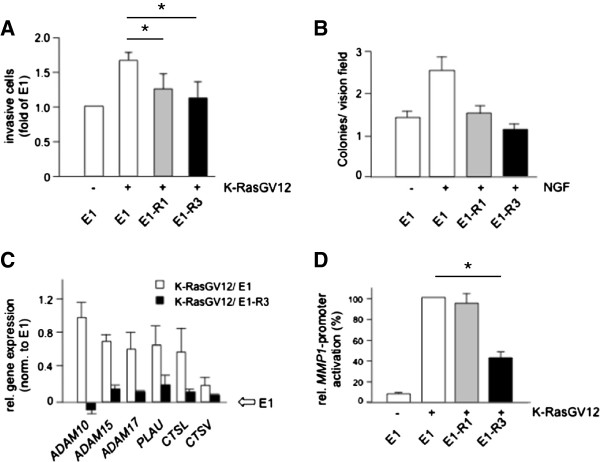
**MSOR mitigate different parameters associated with cellular transformation. (A)** The influence of mono- and trivalent wild-type RBDs on Ras-driven invasion was analyzed after transient transfection of COS-7 cells with expression constructs for K-RasG12V and E1, E1-R1 or E1-R3 and subsequent transmigration of transfected cells through a Matrigel® layer. The figure shows the average of three independent experiments. **(B)** The impact of mono- and trivalent wild-type RBD probes on c-Met-stimulated anchorage-independent growth was investigated by seeding NIH3T3-TM cells transiently expressing E1, E1-R1 or E1-R3 into soft agar and subsequent culture in the presence or absence of 25 ng/ml NGF. Colony formation was evaluated by counting of colonies in at least ten arbitrarily selected vision fields. The figure shows the average of three independent experiments. **(C)** Effect of the trivalent wild-type RBD construct on the Ki-RasG12V-induced protease gene expression. COS-7 cells were transiently transfected with a plasmid encoding E1 alone or an expression construct for K-RasG12V along with E1 or E1-R3. The impact of RBD constructs on K-RasG12V-stimulated expression of different proteases was analyzed on a custome oligonucleotide microarray. Signals were assessed densitometrically and normalized to the E1 expression level. See Material and methods for a more detailed description. Data are derived from three independent experiments. **(D)** Consequences of mono- and trivalent wild-type RBD probes on the K-RasG12V-stimulated induction of the human *MMP1*-promoter. NIH3T3 cells were transiently transfected with E1, E1-R1 or E1-R3 together with an expression construct encoding K-RasG12V as indicated. Then, a *MMP-1*-firefly-luciferase reporter plasmid was co-transfected along with a reference renilla luciferase construct and the relative luciferase activity was determined. The figure shows the average of three independent experiments each performed in duplicates.

Oncogenic Ras-signaling stimulates several pro-tumorigenic pathways that regulate cell proliferation, migration and invasion, among other events. Given their ability to inhibit K-RasG12V-signaling, we hypothesized that MSOR might block aspects of oncogenic Ras-driven transformation. First, we tested the ability of E1-R1 and E1-R3 to block K-RasG12V-induced invasion in matrigel. As shown in Figure 2A, both wild-type RBD-variants interfered with the K-RasG12V-induced invasion of COS-7 cells in matrigel-coated trans-well migration chambers. Secondly, we investigated whether MSOR would also affect anchorage-independent growth, another important hallmark of cellular transformation. To this end we chose to study NIH3T3 cells, since these cells retain numerous features of untransformed cells including cell-cell contact inhibition or the requirement for substrate attachment for productive growth and proliferation. However, NIH3T3 cells do not express EGFR, the prototypical receptor tyrosine kinase commonly used to robustly activate Ras [43], but instead express high levels of PDFGR which is a poor Ras activator. To study Ras signaling in these cells we employed an engineered subline termed NIH-TM which responds to stimulation with Nerve Growth Factor (NGF) owing to the stable expression of a TrkA/c-Met hybrid receptor composed of the extracellular part of Trk and the intracellular domain of c-Met [44]. Stimulation of c-Met activates Ras via the canonical Grb-2/Sos pathway and induces proliferation of NIH3T3 cells [45]. Moreover, over-activation of this receptor tyrosine kinase promotes tumor growth and metastasis [46]. Accordingly, NGF-treatment of NIH-TM cells lead to increased colony formation in soft agar and this effect was completely reversed in the presence of E1-R1 or E1-R3 (Figure 2B), consistent with the ability of wild-type RBD-constructs to also block growth factor-stimulated Ras signaling.

Anchorage-independent growth and cell invasion depend on the action of matrix-degrading enzymes. The promoter region of several protease-encoding genes contains a Ras-responsive element (RRE) or an RRE-like enhancer motif [47,48]. Microarray analysis confirmed that oncogenic K-Ras induced the expression of several protease genes of the ADAM’s and cathepsin families that act both intra- and extracellularly and are involved in matrix remodeling (Figure 2C, Additional file 1). Importantly, the Ras-stimulated upregulation of these proteases was abrogated by E1-R3 (Figure 2C, Additional file 1). Furthermore, this MSOR-construct decreased RasG12V-dependent activation of the RRE-containing *MMP-1* promoter in NIH3T3 cells, as assayed using a luciferase reporter system (Figure 2D). Interestingly, in this case the single RBD unit (E1-R1) was unable to even partially inhibit the effect of K-RasG12V (Figure 2D) or H-RasG12V (Additional file 2), highlighting once more the oligomerization dependent, adjustable blocking potency of MSOR. Moreover, these data suggested that distinct end points of oncogenic Ras signaling exhibit varying sensitivities to the action of RBD polypeptides.

### MSOR interfere with Ras-dependent cell survival signaling and induce apoptosis

**Figure 3:**
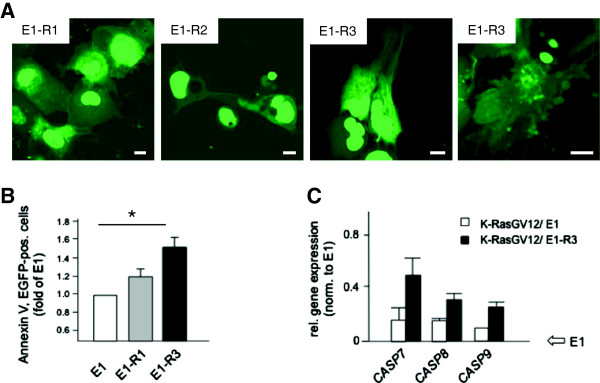
**Targeting endogenously active Ras by MSOR impacts on cell survival. (A)** Confocal images of Cos-7 cells transiently expressing EGFP or mono-, di- or trivalent wild-type RBD probes. Scale bar 10 μm. **(B)** The fraction of dead cells among E1-, E1-R1- or E1-R3-expressing cells was determined by measuring Annexin V-positive cells using FACS and normalized to the EGFP-expressing condition. Data represent three independent experiments. **(C)** Caspase gene expression in COS-7 cells, transiently transfected with an expression construct for K-RasG12V along with E1 or E1-R3 was analyzed on a custom oligonucleotide microarray. Signals were assessed densitometrically and normalized to the E1 expression level. See Material and methods for a more detailed description. Data are derived from three independent experiments. See Material and methods for a more detailed description.

So far, the impact of MSOR was studied in the context of oncogenic Ras signaling. However, we noticed previously that expression of high affinity MSOR in the absence of constitutively active Ras has a profound effect on the morphology and viability of various types of cells [38]. Figure 3A shows fluorescence images of COS-7 cells expressing E1-R1, E1-R2 or E1-R3 in the absence of Ras co-transfection. Whereas expression of E1-R1 had no obvious effect on morphology and overall appearance of COS-7 cells, expression of the more avid MSOR variants E1-R2 and E1-R3 induced dramatic changes in cell morphology giving rise to spindle-like and asymmetric shapes, fragmented nuclei, vacuoles and membrane blebbing (Figure 3A).

Since membrane blebbing and other phenotypic changes in cells expressing E1-R3 were reminiscent of apoptotic cells we investigated whether or not MSOR induced apoptosis of cells expressing native wild-type Ras. Annexin V-staining confirmed the increased occurrence of apoptosis among MSOR-transfected COS-7 cells (Figure 3B). These data are compatible with a MSOR-mediated blockade of basal, endogenous Ras-GTP signaling, which reportedly protects cells from apoptosis [49]. This notion was further supported by microarray data showing that E3-R3 upregulated the expression of caspases (Figure 3C, Additional file 1), even so in the presence of co-transfected oncogenic Ras. Importantly, the higher potency of E1-R3 *versus* E1-R1 in apoptosis induction was not a result of an overall higher total number of RBD units but caused by the presence of the oligovalent polypeptides, because cells expressing up to 5 fold higher levels of E1-R1 did not exhibit the same signs of cellular breakdown (unpublished observation). We concluded from these findings that MSOR impair cell survival by the sustained strong sequestration and blockade of basal Ras-GTP signaling.

### Adjusted inhibition of Ras-mediated cellular effects by inducible MSOR expression

**Figure 4:**
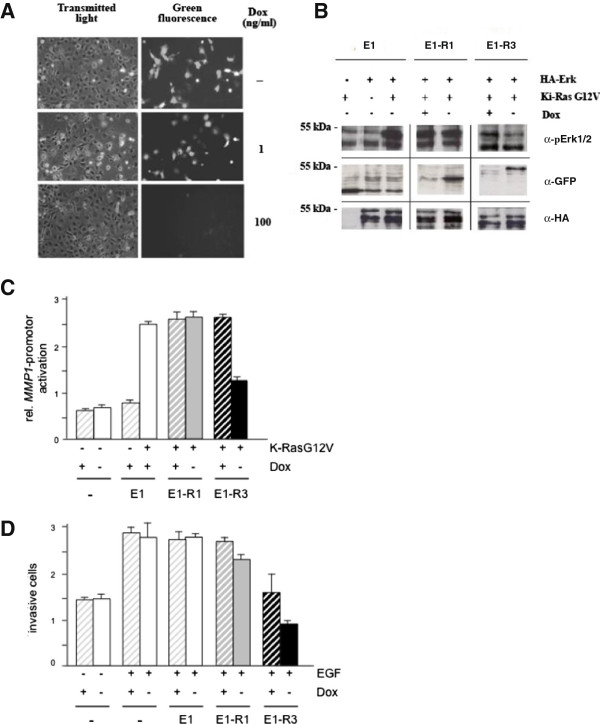
**Adjusted, low-level expressed MSOR maintain their Ras-blocking activity. (A)***Tet-off* promoter-controlled expression of RBD-monomers and MSOR. COS-7 cells were transfected with pNRTIS 12-derived plasmids encoding E1-R3 and subsequently left untreated or treated with different concentrations of doxycycline (Dox) to modulate MSOR expression levels. **(B)** The impact of inducible mono- and trivalent wild-type RBD probes on oncogenic Ras-stimulated MAP-kinase signaling. COS-7 cells were transiently transfected with different constructs as indicated. Next, cells were divided into samples that were grown either in the absence (-) or presence (+) of 100 ng/ml Dox. Cells lysates were analyzed by Western blot detecting phosphorylated and total Erk2 and the expression of EGFP-constructs. One representative experiment out of three independent experiments is shown. **(C)** Influence of inducible mono- and trivalent wild-type RBD probes on oncogenic Ras-stimulated *MMP-1* promoter activation. NIH3T3 cells were transiently transfected with the *MMP-1*-luciferase reporter, the K-RasG12V-encoding construct and Tet-off-controlled EGFP-constructs E1, E1-R1 or E1-R3. Expression of EGFP-constructs was turned on (-Dox) or off (+100 ng/ml Dox) and reporter gene activity was measured. The figure shows the average result from three independent experiments each performed in triplicate. **(D)** Influence of inducible mono- and trivalent wild-type RBD probes on EGF-stimulated cell invasion. COS-7 cells were transfected with the Tet-off-regulated EGFP-constructs indicated, and cultured in the absence of Dox. Subsequently, EGFP-expressing cells were collected by preparative fluorescence activated cell sorting and cultured in absence or presence of 100 ng/ml Dox to regulate the expression of E1, E1-R1 and E1-R3. Then cells were collected, seeded onto Matrigel-coated Transwells and subjected to invasion in absence or presence of 50 ng/ml EGF. Results represent the means of two entirely independent experiments.

The cytotoxic effects of E1-R2 and E1-R3 prompted us to develop strategies that allowed tuning the action of MSOR. First, we employed a tetracycline controllable system (*Tet-off* system) to regulate the expression of highly avid MSOR like E1-R3. COS-7 cells were transiently transfected with Tet-off constructs driving the expression of monomeric E1-R1 and trimeric E1-R3. In a non-repressed setting, expression of E1-R1 and E1-R3 was readily detectable (Figure 4A) but did not induce the prominent morphological changes observed under conditions of enhanced expression (Figure 3A). Addition of increasing concentrations of the tetracycline-derivative doxycycline (Dox) to the culture medium inhibited the MSOR expression in a concentration-dependent manner (Figure 4A), thus confirming the proper function of the inducible expression system.

Next, the effect of experimentally induced expression of RBD-constructs on the RasG12V-stimulated Erk2-activation in COS-7 was assessed (Figure 4B). Induction of E1-R3 expression decreased RasG12V-sparked Erk2-phosphorylation while the corresponding monomer was ineffective under the same conditions. This finding contrasts with the blocking action of E1-R1 in transient overexpression experiments (see Figure 1B) and suggested that MSOR-dependent blockade of distinct Ras elicited effects may depend on the expression levels achieved in individual experiments and/or may sometimes require sustained action of the MSOR proteins over a longer period of time.

In agreement with its blocking of Erk2 activation, the wild-type trimer but not the monomer was able to blunt RasG12V-stimulated activation of the *MMP-1*-reporter in NIH3T3 cells (Figure 4C) and EGF-driven invasion of COS-7 cells (Figure 4D).

Taken together these data illustrate the efficacy of inducible MSOR to control and tune Ras action.

### Controlled inhibition of oncogenic Ras by attenuated MSOR

Another potential approach for reducing the cytotoxicity of MSOR constructs was the introduction of specific mutations in the RBD that strongly decrease their affinity for Ras-GTP, like the R59A mutation described above. This approach was successfully applied previously, and lead to the development of the double point mutant RBD-R59A/N64D, which in its trimeric form E1-R3(A/D) retained high avidity for Ras-GTP while exhibiting little cytotoxicity [38,39].

**Figure 5:**
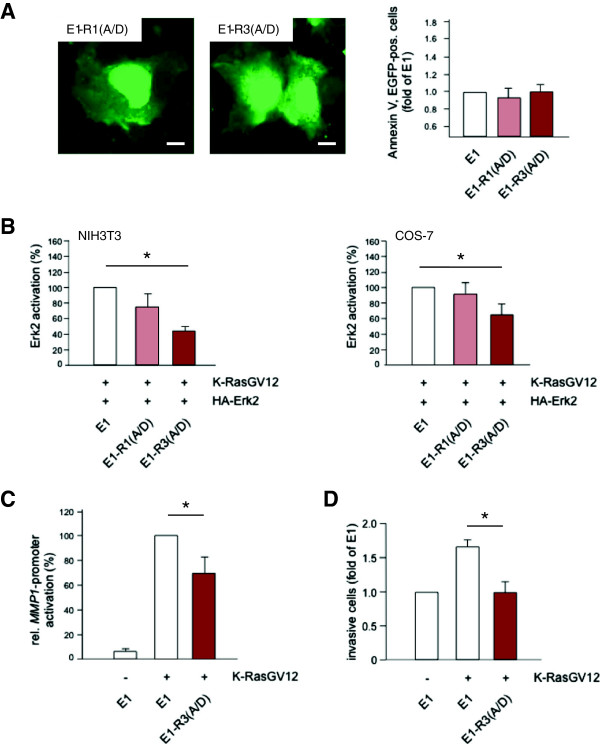
**A non-toxic MSOR-variant efficiently blocks Ras-induced signaling and transformation. (A)** Confocal images of COS-7 cells transiently expressing the mono- and trivalent low affinity RBD-R59A/N64D probes (*left panel*). Scale bar 10 μm. Cell death analysis of COS-7 cells transiently expressing E1, E1-R1(A/D) or E1-R3(A/D) using Annexin V-staining (*right panel*). The average of three independent experiments is shown. **(B)** Quantitative assessment of Western blot analysis of oncogenic Ras-induced Erk2 activation. NIH3T3 (*left panel*) and COS-7 (*right panel*) cells transiently expressing K-RasG12V, HA-tagged Erk2 and E1, E1-R1(A/D) or E1-R3(A/D) were subjected to western blot analysis detecting phosphorylated and total Erk2. Signals were quantified and expressed as ratio of phosphorylated and total Erk2. The ratios derived from the different conditions were normalized to EGFP, and the average of three independent experiments is shown in the figure. **(C)** Impact of low-affinity RBD constructs on the K-RasG12V-stimulated induction of the human *MMP1*-promoter. NIH3T3 cells were transiently transfected with the different RBD-constructs indicated. Then, a *MMP-1*-firefly-luciferase reporter plasmid was co-transfected along with a reference renilla luciferase construct and the relative luciferase activity was determined after 24 h. The figure shows the result of three independent experiments each performed in duplicate. **(D)** Influence of the low-affinity RBD probes on the oncogenic Ras-stimulated invasiveness of COS-7 in a Matrigel-coated Transwell assay. The data depicted show the average from three independent experiments.

In line with those features, over-expression of E1-R3(A/D) or its monomeric counterpart E1-R1(A/D) in COS-7 cells did not induce morphological changes or apoptosis (Figure 5A) as observed with the wild-type MSOR E1-R3 (Figure 3A). Similarly to E1-R1(A), the E1-R1(A/D) monomer did not impact on oncogenic K-Ras-driven signal transduction (Figure 5B). However, the trivalent double point mutant E1-R3(A/D) clearly diminished the RasG12V-induced Erk2-activation in both COS-7 and NIH3T3 cells (Figure 5B). Moreover, E1-R3(A/D) did also abrogate aspects of cellular transformation such as *MMP1*-activation (Figure 5C) and cell invasion (Figure 5D). Collectively, these findings illustrated that even low-affinity, biologically inert modules like the double point mutant RBD-R59A/N64D can be converted into robust scavengers of oncogenic Ras by increasing their avidity for Ras-GTP via oligomerization.

## Discussion

This study describes a novel application for the RBD of c-Raf as a building block of multivalent probes for the adjustable and graded inhibition of oncogenic Ras signaling. The data presented herein illustrate that MSOR are able to specifically target and block various events downstream of aberrant Ras-signaling including Erk-activation (Figures 1B and 5B), induction of matrix-remodeling enzymes (Figures 2C, 2D and 5C), Ras-stimulated matrix invasion (Figures 2A and 5D) and growth factor-induced contact-independent growth (Figure 2B). Moreover, it is worth emphasizing that MSOR not only counteracted the action of oncogenic Ras itself but also abrogated several parameters of cellular transformation sparked by cell surface growth factor receptors that signal via Ras (Figures 2B and 4D), suggesting a potentially broader application of MSOR in pro-tumorigenic settings that involve aberrant Ras-signaling. Importantly, the binding properties of MSOR are amenable to manipulation at three different levels: 1^st^, by varying their oligomerization grade and thus the avidity towards Ras-GTP [38], 2^nd^, by introducing point mutations in single RBD modules, affecting the affinity of individual RBDs to Ras-GTP and 3^rd^ by regulating their protein expression levels. Several observations reported here strongly indicate that different combinations of the three parameters enumerated above will generate MSOR with distinct binding and inhibitory properties. For example, the wild-type RBD-monomer R1 effectively blocked different aspects of enhanced Ras-signaling (Figures 1B, 2A and 2B) when over-expressed to high levels in cells but it was ineffective at low expression levels in most cell types studied (Figure 4). In contrast, the trivalent protein R3 exhibited strong inhibitory effect in the same settings irrespective of its expression levels, suggesting that a higher avidity for Ras-GTP effectively increases the blocking potency and essentially compensates for low expression levels.

Along the same lines, we observed that one and the same RBD probe exhibits variable potencies for blocking different events downstream of oncogenic Ras. For instance, the monovalent wild-type unit R1 does not even partially affect matrix metalloproteinase induction by RasG12V, even though it does impinge on proximal Ras effectors like Erk in essentially the same system. The simplest explanation for this and related observations is that distinct cell biological readouts of oncogenic Ras require the action of different Ras effector pathways, or combinations thereof, that are distinctively sensitive to MSOR action. Indeed, the three most well characterized Ras effectors, Raf, PI3K and Ral GDS exhibit a large variance in their thermodynamic affinities for Ras-GTP of up to two orders of magnitude [50]. Taking into account that many other parameters such as steric considerations or subcellular compartmentalization aspects can additionally regulate Ras/effector coupling *in vivo*, it is well conceivable that the engagement of different effectors by oncogenic Ras may be distinctively sensitive to MSOR action. Indeed, in the mentioned case of *MMP-1* regulation by oncogenic Ras, available evidence suggest that *MMP-1* expression requires other Ras-sparked signals in addition to Erk, including activation of p38α and likely others [51]. Alternatively, the partial only blockade of a Ras effector pathway like the Raf/MEK/Erk cascade may not suffice to compromise all-or-nothing, switch-like type of threshold-controlled processes [52,53]. Furthermore the final outcome to Ras/Erk pathway activation is subject to regulation by intrincate, as yet not fully understood positive and negative feedback loops [54-56] that may add further levels of complexity in settings of incomplete Ras-GTP blockade by MSOR. Taken together, these considerations indicate that the degree of MSOR-mediated inhibition of a proximal downstream effector of Ras such as Erk, will not necessarily translate into the same degree of inhibition of a given Ras-dependent tumorigenic hallmark. At the same time, from a methodological point of view, these considerations indicate that beyond their use as blockers of Ras signaling, MSOR can be instrumental tools for delineating the regulatory and mechanistic properties of the signaling network downstream of Ras.

As mentioned before, the affinity of the individual RBD modules for Ras-GTP is one major parameter that allows adjusting the strength of binding and inhibition. Many RBD point mutants have been described and extensively characterized biochemically and structurally with regard to their interaction with Ras-GTP. For example, replacing arginine 59 for alanine in RBD yields a polypeptide with 29-fold diminished affinity for Ras-GTP, and incorporation of a second mutation (N64D) further reduces affinity by a factor of four [14,16]. In agreement with those properties, the single R59A and double R59A/N64D mutants did not block any of the investigated Ras effects if applied in their monomeric forms (R1(A) and R1(A/D)) but they did inhibit Ras-GTP signaling at all investigated levels once converted to their trivalent counterparts R3(A) and R3(A/D) (Figures 1 and 5). This was a striking observation since it evidenced that even RBD mutants deemed to be biologically inert due to negligible Ras-GTP binding could turn into potent Ras blockers if rendered more avid towards Ras-GTP by oligomerization. These considerations gain further relevance in the light of recent insights into the Ras-dependent activation mechanism of Raf. A wealth of experimental data has recently established that Raf kinases function as homo- and heterodimers [57-60]. Although many details of Raf regulation remain obscure it is evident that only the dimeric form is responsive and sensitive to activation by Ras-GTP [57]. Thus, the oligomeric RBD-based units, as used in the present study may, in essence, reflect and recapitulate aspects of the physiological interaction of Ras-GTP with a Raf dimer.

Aberrant Ras activity due to oncogenic mutations is found with high frequency in different human malignancies and remains one of the most attractive molecular targets for rational cancer treatment [15]. Although different approaches such as DNA vaccination, microRNA targeting Ras and farnesyl-transferase inhibition have been exploited as putative therapeutic strategies to block oncogenic Ras, they have all not stood the test of time and clinical trials [61]. More recently, various novel structure-guided approaches for targeting oncogenic Ras have been described [29,33,35]. Of note, others have previously exploited the single RBD from c-Raf-1 or other Ras-GTP interacting protein modules in order to suppress oncogenic Ras-induced cell transformation in various experimental settings [62,63]. The MSOR approach described here adds to this panel of Ras inhibitory strategies. As a unique feature, MSOR are amenable to fine-tuning for adjustment of their inhibitory strength. Their potent effect on different parameters of Ras-stimulated cellular transformation *in vitro* (Figures 2, 4 and 5) provides a solid basis for further studies investigating the performance of MSOR in the context of *in vivo* tumor growth and progression. However, being genetically encoded, the use of MSOR for treatment of Ras-dependent tumours must await improved gene delivery protocols. Alternatively, however, MSOR could potentially be delivered via alternative routes, taking advantage of specific features of Ras-driven tumours. For example, Ras-positive tumours exhibit strongly enhanced macropinocytosis [64], a property that could be exploited to selectively deliver polypeptides, nanoparticles or other types of drugs into the tumour cells.

## Conclusions

The data presented herein introduce the *m*ultivalent *s*cavengers of *o*ncogenic *R*as (MSOR) that can be applied as versatile, adjustable Ras-GTP selective probes. MSOR represent novel tools to potently inhibit the action of oncogenic Ras and can be employed in basic research studies of oncogenic Ras function and studies aiming to block tumor growth and progression.

## Material and methods

### Cell lines, transfection

COS-7 cells and NIH3T3 cells were obtained from the DSMZ (German Collection of Microorganisms and Cell Cultures, Braunschweig, Germany) and cultured in DMEM medium supplemented with 10% FCS and 100 μg/ml Gentamycin. Transfection of COS-7 and NIH3T3 cells with plasmid DNA was performed with Nucleofection^R^ employing a Nucleofector^R^ device, “Solution V” and “Program A24” according to directions of the manufacturer (Lonza, Cologne, Germany) or using the Polyfect™ transfection reagent following the directions of the manufacturer (Qiagen, Hilden, Germany).

### DNA constructs

Expression constructs for EGFP-fused RBD-mono- and oligomers based on the EGFP-C2 vector (Clontech, Mountain View, CA, USA) as well as plasmids encoding constitutively active RasG12V mutants and HA-tagged Erk2 have been described previously [38,39]. Inducible expression constructs for EGFP and EGFP-MSOR were generated on the basis of the bicistronic *Tet-off* vector pNRTIS-21 [38]. cDNAs encoding EGFP and EGFP-RBD fusions were subcloned as *EcoR*I/*Not*I fragment into pNRTIS-21 by standard molecular biology procedures. The luciferase reporter gene plasmid containing the human *MMP-1* promoter has been described previously [65].

### Inducible MSOR expression

COS-7 cells were transiently transfected with constructs encoding inducible, EGFP, mono- or oligovalent EGFP-RBD probes. Expression of these constructs was induced or repressed by culturing the cells in absence or presence of 100 ng/ml Doxycyclin, respectively. Fluorescence microscopy demonstrated that the expression of EGFP-constructs was efficiently suppressed in cultures exposed to Doxycyclin for 72 h.

### Fluorescence microscopy

Visualization of EGFP fluorescence was performed with an Axiovert 135 M fluorescence microscope (Carl Zeiss GmbH, Jena, Germany).

### Western blot analysis

Western blot analysis of cell lysates for protein expression and/or protein phosphorylation has been previously described in detail [38].

### Luciferase reporter gene assay

5 × 10^5^ NIH3T3 cells were grown in six-well plates (Greiner, Frickenhausen, Germany) in 2 ml DMEM/10% FCS to 80–90% confluency. Cells were transferred to 1 ml of fresh medium and transfected with plasmids encoding oncogenic Ras and EGFP-coupled RBD-probes. The next day, cells were transfected simultaneously with 1 μg *firefly* luciferase-coupled *MMP-1*-promoter construct, *MMP-1-2G*/pGL3 [65] and 0.1 μg pRL-TK plasmid encoding *renilla* luciferase (Promega, Madison, WI). 14 h post transfection, cells were harvested using “reporter lysis buffer” (Promega). *Firefly* and *renilla* luciferase activities were determined using the Dual-Luciferase Reporter Assay System kit (Promega, Madison, WI, USA) following the manufacturer’s instructions. Luminescence was measured using the Promega GLOMAX^R^ 96 Luminometer and reported as relative light units. Relative *MMP-1*-promoter activation was derived by normalizing the *firefly* luciferase activity to *renilla* luciferase activity.

### Soft agar colony formation assay

The soft agar assay to analyze the anchorage independent growth of NIH3T3-TM cells was performed as described before [44]. Briefly, NIH3T3-TM cells, were transfected with constructs encoding EGFP or EGFP-RBD probes. Subsequently, 2 × 10^4^ transfected cells were suspended in 0.5 ml DMEM/10% FCS supplemented with 0.4% Seaplaque agarose and seeded per well of a 24-well tissue culture plate (Greiner) on a layer of 0.5 ml DMEM/0.8% Seaplaque agarose. Cultures were fed with 0.2 ml of DMEM/10% FCS in the presence or absence of 25 ng/ml NGF every 3 days for 2 weeks. Colonies were then stained with p-iodonitrotetrazolium violet (Sigma, Munich, Germany) and microscopically inspected. Data are derived from counting the number of colonies in at least ten arbitrarily selected vision fields.

### Protease expression analysis by cDNA arrays

cDNA microarrays of protease and protease inhibitor sequences on nylon membranes and the synthesis of digoxigenin labeled cDNA have been described previously [66]. Detailed information on the generation of the protease/protease inhibitor probes, their arrangement on the membranes as well as experimental details have been published [67]. In brief, cDNA prepared from COS-7 cells was digoxigenin-labeled and hybridized on a custom oligonucleotide microarray comprising housekeeping genes, positive and negative controls, and genes representing a collection of human intra- and extracellular proteases, and protease inhibitors. Hybridization patterns were subsequently detected by chemiluminescence and analyzed using the AIDA imaging software (Raytest, Straubenhardt, Germany). Average densitometry signals of duplicate spots from K-RasG12V/E1- and K-RasG12V/E1-R3-xpressing cells were corrected for the background and normalized against the respective signal from E1-expressing cells.

### Cytometric cell analysis and sorting

Cytometric measurements and cell sorting was performed using a FACS Calibur^R^ instrument (BD Biosciences, Heidelberg, Germany) equipped with a 488 nm laser and the CellQuestPro^R^ software. For flow cytometric analysis of EGFP expression, cells transfected with constructs encoding EGFP or EGFP-RBD probes were trypsinized and adjusted to a density of 1 × 10^6^/100 μl, forward scatter (cell size) and sideward scatter (cell granularity) were determined and vital cells were gated. EGFP signals were recorded using a 515–545 nm filter and plotted against the number of events. Sorting of EGFP-positive cells was performed following transfection of 2 × 10^6^ COS-7 cells with pRNTIS 21-derived expression constructs encoding EGFP or EGFP-RBD-probes and subsequent cultivation of cells for 48 h. This procedure routinely yielded an enrichment of EGFP-expressing cells to approximately 90%.

### Annexin V staining

COS-7 cells were grown in six-well plates (Greiner) to 80% confluency, transfected the next day with plasmids encoding EGFP or EGFP-coupled RBD-probes and then cultured for additional 24 h in fresh culture medium. Cells were detached by trypsin/versene (Gibco®/Life Technologies, Darmstadt, Germany) and collected by centrifugation. The cell pellet was washed twice in 1 × PBS and re-suspended in 220 μl 1 × bindings buffer (BD Biosciences). The sample was divided in two: 100 μl sample were left untreated, the other 100 μl were supplemented with 2.5 μl Annexin V-APC (BD Biosciences). The different preparations were incubated for 5 min at 37°C and then for 25 min at room temperature in the dark. To determine the proportion of dead cells among the EGFP or EGFP-RBD-expressing COS-7 cells Annexin V-APC was measured using the FACS Calibur^R^ instrument (BD Biosciences) and plotted against EGFP. Subsequent propidium iodide (*Merck Biosciences*, *Schwalbach*, *Germany*) staining revealed that approximately 85% of the transfected, dead cells underwent apoptosis.

### In vitro cell invasion assay

COS-7 invasion was studied using polycarbonate Trans-wells (Corning Costar Corp., Cambridge, MA, USA) as previously described [44]. Briefly, cells were cultured in DMEM supplemented with 10% FCS and, optionally, 100 ng/ml Doxycyclin and/or 50 ng/ml EGF for 72 h. 2 × 10^5^ cells were then seeded onto membrane filters coated with Matrigel® (BD Biosciences) and transmigration through the Matrigel® layer was determined after incubation for 24 h. Cell invasion was expressed as the average number of migrated cells per vision field (100× magnification) of at least seven, arbitrarily selected vision fields.

### Statistics

All data are expressed as the mean S.E.M. SPSS for Windows was used for all statistical analyses. The non-parametric Mann–Whitney (U) test and one-way ANOVA with Newman-Keuls Multiple Comparisons were used to analyze if differences among different experimental groups are statistically significant (p < 0.05).

## Abbreviations

ADAM: A disintegrin and metalloprotease domain; CASP: Caspase; CTSL: Cathepsin L; CTSV: Cathepsin V; DMEM: Dulbecco’s modified eagle medium; Dox: Doxycycline; EGF: Epidermal growth factor; EGFP: Enhanced green fluorescent protein; Erk: Extracellular signal regulated kinase; FACS: Fluorescence activated cells sorting; FCS: Fetal calf serum; GAP: GTPase activating protein; GEF: Guanosine nucleotide exchange factor; HA: Haemagglutinin; MAP: Mitogen-activated protein; MMP-1: Matrix metalloproteinase-1; MSOR: Multivalent scavengers of oncogenic Ras; NGF: Nerve growth factor; RBD: Ras-binding domain; RRE: Ras-responsive element; tTA: Tetracyline-regulated transactivator; PLAU: Plasminogen activator, urokinase.

## Competing interests

The authors declare to have no competing interests.

## Authors’ contributions

MA planned and performed experiments. AB performed experiments. RS provided essential contributions to fluorescent activated cell sorting. IR and KF designed and supervised the study. MA, KF and IR wrote the manuscript. All authors read and approved the final manuscript.

## Supplementary Material

Additional file 1 **MSOR inhibit oncogenic Ras-stimulated gene expression. (A)** Representative pictures of a custome oligonucleotide microarray covering various proteases and integrins that demonstrate differential effects of the MSOR E1-R3 on K-RasG12V-stimulated gene expression in COS-7 cells. **(B)** Graphic presentation of K-RasG12V/E1-regulated genes that were either induced or repressed compared to E1-expressing COS-7 cells and counteracted by E1-R3. Up- and down regulation of gene expression is depicted in green and red, respectively. Click here for file

Additional file 2 **MSOR block oncogenic H-Ras-induced signaling.** NIH3T3 cells were transiently transfected with E1, E1-R1 or E1-R3 together with an expression construct encoding H-RasG12V as indicated. Subsequently, plasmids encoding an *MMP-1*-firefly-luciferase reporter and renilla luciferase were co-transfected along and the relative luciferase activity was determined. The figure shows the average of three independent experiments each performed in duplicates. Click here for file
