# Supplementary material for: Graded inhibition of oncogenic Ras-signaling by multivalent Ras-binding domains
Source: Cell Commun Signal. 2014 Jan 2;12:1. doi: 10.1186/1478-811X-12-1 (PMC3898410; doi:10.1186/1478-811X-12-1)
Supplement: Additional file 1 — MSOR inhibit oncogenic Ras-stimulated gene expression. (A) Representative pictures of a custome oligonucleotide microarray covering various proteases and integrins that demonstrate differential effects of the MSOR E1-R3 on K-RasG12V-stimulated gene expression in COS-7 cells. (B) Graphic presentation of K-RasG12V/E1-regulated genes that were either induced or repressed compared to E1-expressing COS-7 cells and counteracted by E1-R3. Up- and down regulation of gene expression is depicted in green and red, respectively. [file 1478-811X-12-1-S1.ppt]

## Slide 1
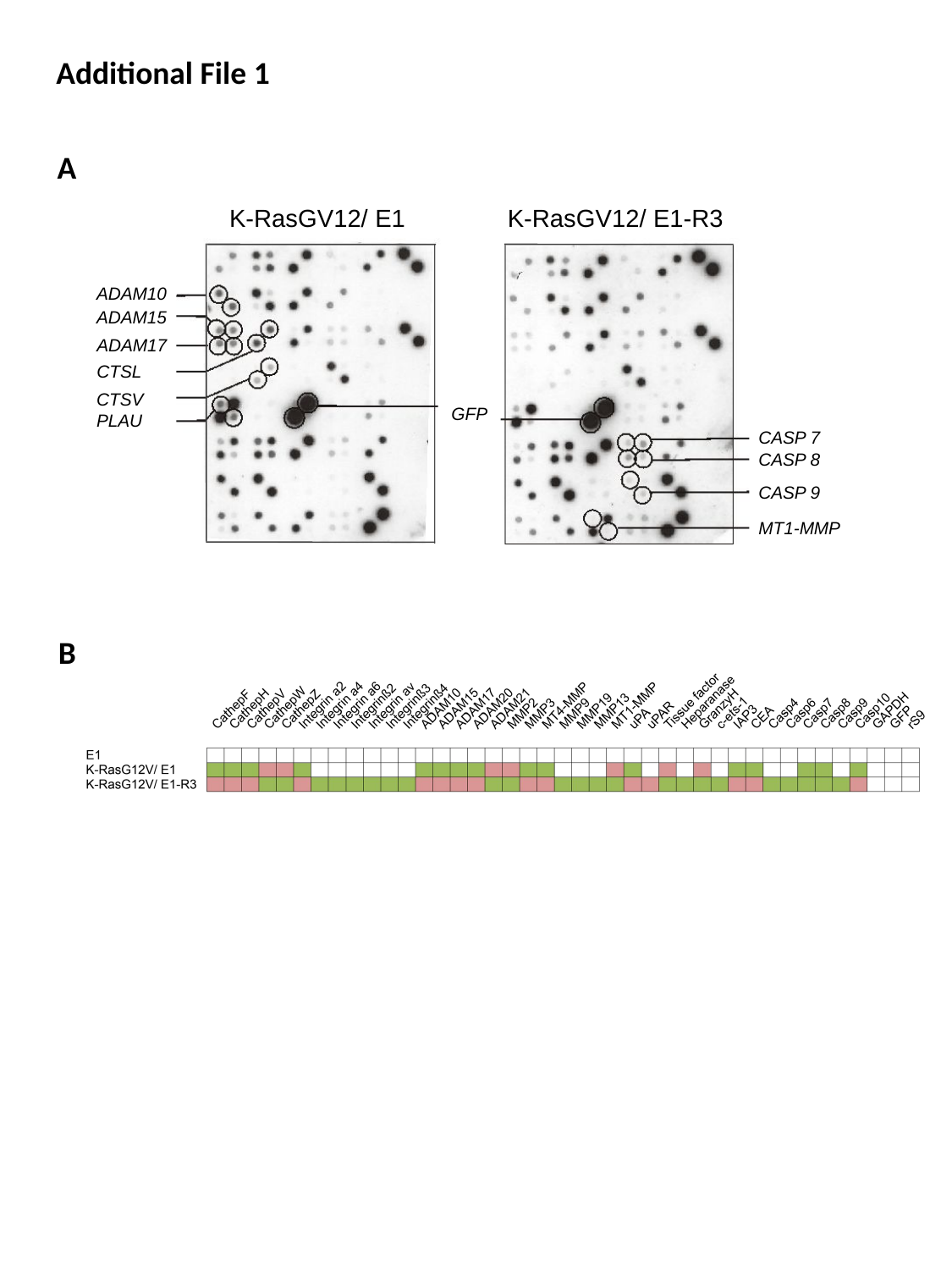

Additional File 1
A
K-RasGV12/ E1
K-RasGV12/ E1-R3
GFP
ADAM10
ADAM15
ADAM17
CTSL
CTSV
PLAU
CASP 7
CASP 8
CASP 9
MT1-MMP
B
