# Supplementary figures and images for: Graded inhibition of oncogenic Ras-signaling by multivalent Ras-binding domains
Source: Cell Commun Signal. 2014 Jan 2;12:1. doi: 10.1186/1478-811X-12-1 (PMC3898410; doi:10.1186/1478-811X-12-1)

## Slide 1
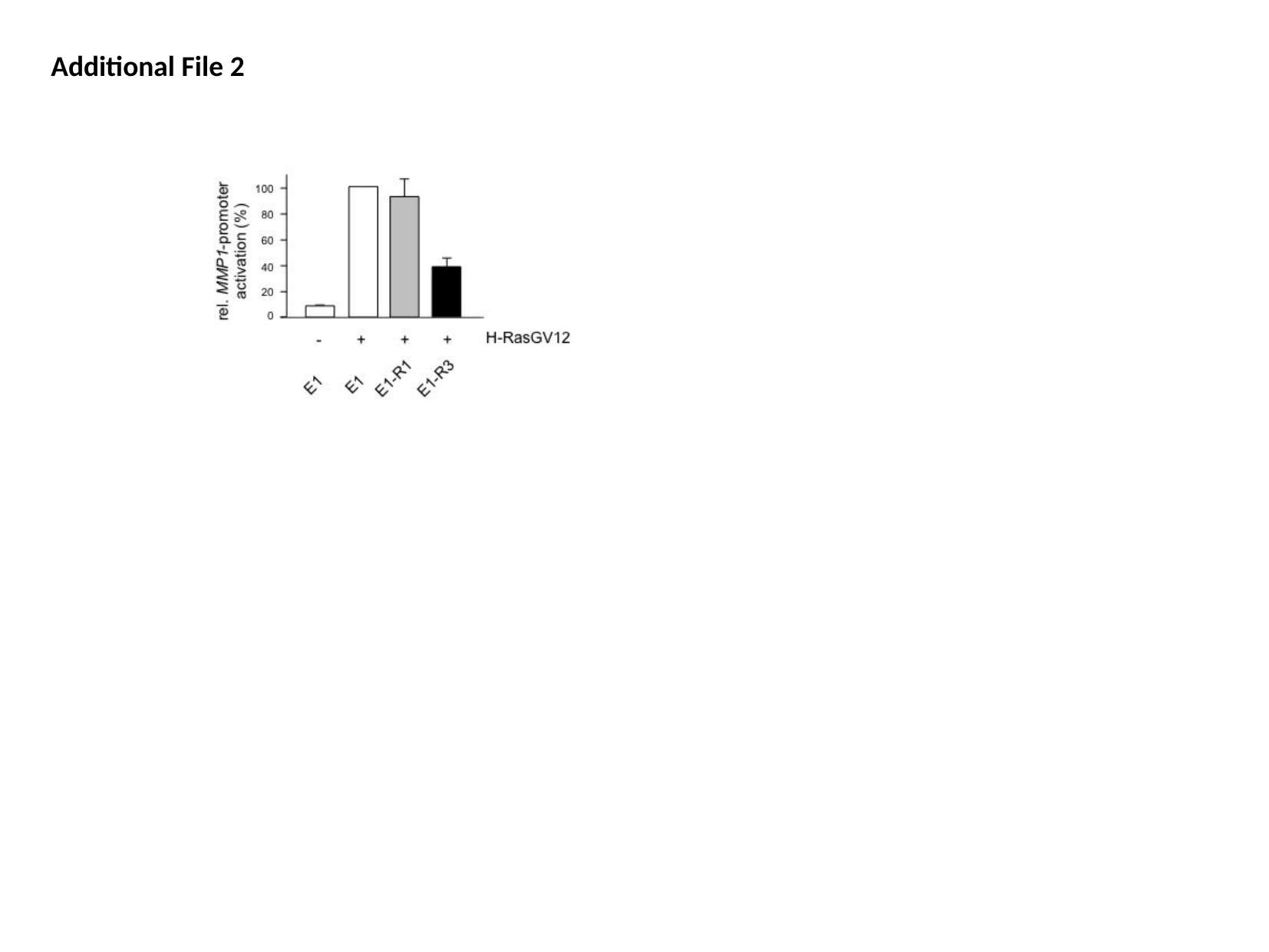

Additional File 2

Supplement: Additional file 2 — MSOR block oncogenic H-Ras-induced signaling. NIH3T3 cells were transiently transfected with E1, E1-R1 or E1-R3 together with an expression construct encoding H-RasG12V as indicated. Subsequently, plasmids encoding an MMP-1-firefly-luciferase reporter and renilla luciferase were co-transfected along and the relative luciferase activity was determined. The figure shows the average of three independent experiments each performed in duplicates. [file 1478-811X-12-1-S2.ppt]
